# Mechanisms of enhanced aggregation and fibril formation of Parkinson’s disease-related variants of α-synuclein

**DOI:** 10.1038/s41598-022-10789-6

**Published:** 2022-04-26

**Authors:** Takashi Ohgita, Norihiro Namba, Hiroki Kono, Toshinori Shimanouchi, Hiroyuki Saito

**Affiliations:** 1 Department of Biophysical Chemistry, Kyoto Pharmaceutical University, 5 Misasagi-Nakauchi-cho, Yamashina-ku, Kyoto, 607-8414 Japan; 2 Graduate School of Environmental and Life Science, Okayama University, Okayama, 700-8530 Japan

**Keywords:** Kinetics, Biophysical chemistry, Protein aggregation

## Abstract

Aggregation of α-synuclein (α-syn) into amyloid fibrils is closely associated with Parkinson’s disease (PD). Familial mutations or posttranslational truncations in α-syn are known as risk factor for PD. Here, we examined the effects of the PD-related A30P or A53T point mutation and C-terminal 123–140 or 104–140 truncation on the aggregating property of α-syn based on the kinetic and thermodynamic analyses. Thioflavin T fluorescence measurements indicated that A53T, Δ123‒140, and Δ104–140 variants aggregated faster than WT α-syn, in which the A53T mutation markedly increases nucleation rate whereas the Δ123‒140 or Δ104‒140 truncation significantly increases both nucleation and fibril elongation rates. Ultracentrifugation and western blotting analyses demonstrated that these mutations or truncations promote the conversion of monomer to aggregated forms of α-syn. Analysis of the dependence of aggregation reaction of α-syn variants on the monomer concentration suggested that the A53T mutation enhances conversion of monomers to amyloid nuclei whereas the C-terminal truncations, especially the Δ104–140, enhance autocatalytic aggregation on existing fibrils. In addition, thermodynamic analysis of the kinetics of nucleation and fibril elongation of α-syn variants indicated that both nucleation and fibril elongation of WT α-syn are enthalpically and entropically unfavorable. Interestingly, the unfavorable activation enthalpy of nucleation greatly decreases for the A53T and becomes reversed in sign for the C-terminally truncated variants. Taken together, our results indicate that the A53T mutation and the C-terminal truncation enhance α-syn aggregation by reducing unfavorable activation enthalpy of nucleation, and the C-terminal truncation further triggers the autocatalytic fibril elongation on the fibril surfaces.

## Introduction

**Figure 1:**
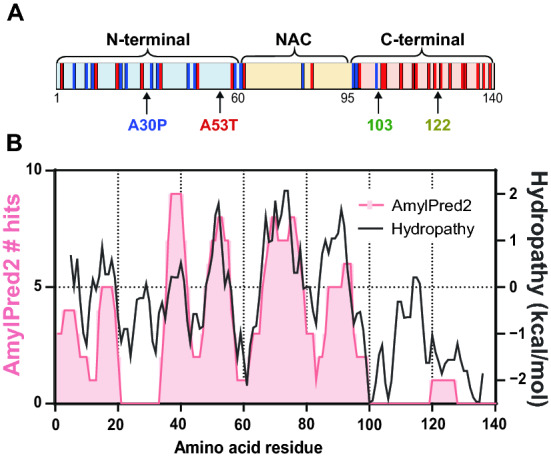
Schematic representation of charge distribution in three regions, aggregation propensity, and hydropathy of α-syn. *A*, Human α-syn consists of three regions: N-terminal (residues 1–60), non-amyloid β component (NAC, residues 61–95), and C-terminal (residues 96–140) regions. The PD-related point mutations and C-terminal truncated sites used in the present study are indicated by arrows. Red and blue lines inside protein domains represent acidic (Asp/Glu) and basic (Lys) amino acid residues, respectively. *B*, Aggregation propensity and hydropathy profiles of α-syn. Aggregation propensity was predicted using the consensus algorithm AmylPred2 (http://aias.biol.uoa.gr/AMTLPRED2/). Hydropathy plot was created using the ExPASy Protscale tool with the Kyte & Doolittle scale (http://web.expasy.org/protscale/).

Parkinson’s disease (PD) is the second most common neurodegenerative disorder characterized by deposition of amyloid fibrils of α-synuclein (α-syn) in the brain^1,2^. α-Syn is an intrinsically disordered, 140-residue protein composed of three domains: positively charged N-terminal region (residues 1‒60), hydrophobic non amyloid β component (NAC) region (residues 61‒95), and negatively charged C-terminal region (residues 96‒140) (Fig. 1A)^3–5^. In physiological neurons, most of α-syn exists as an unstructured monomer, with a part being α-helical state on synaptic vesicles or plasma membranes^4–7^. Recent in-cell NMR study suggests that about 90% of intracellular α-syn transiently interacts with Hsp70 and Hsp90 chaperones, competing with the membrane binding^8^. In pathological conditions, α-syn undergoes a structural transition to β-sheet-rich state, resulting in the formation of toxic oligomers and amyloid fibrils^4,9–11^. These aggregated forms of α-syn are strongly associate with lipids in neuronal inclusions called Lewy bodies or Lewy neurites^12^. During this process, α-syn acquires neurotoxicity and propagation property which induce a progressive loss of neurons and cause PD^13–17^. Once toxic forms of α-syn are generated, it is considered that the autocatalytic recruitment of endogenous α-syn into further pathologic forms happens to cause widespread of toxic α-syn within and between neuronal and glial cells in a prion-like manner^13,18,19^. Therefore, inhibition of aggregation and fibril formation of α-syn is expected to be a potential therapeutic strategy for PD^16,17,20,21^. For rational design of potent amyloid inhibitors for the PD therapy, a detailed understanding of the molecular mechanisms underlying the aggregation and fibril formation of α-syn in pathological conditions is required^22–24^.

Although most cases of PD are sporadic, there are point mutations in the N-terminal region of α-syn that cause familial PD. Currently, eight familial PD mutations (A18T, A29S, A30P, E46K, H50Q, G51D, A53T, and A53E) have been reported^25–32^. A30P and A53T are frequent mutations firstly identified in Italian and German families, respectively, with autosomal dominant inheritance for PD^26,27^. Several mutations including the A30P and A53T are known to lead to early onset of PD, despite large variations in the age of disease onset have been observed^25,33^. The A53T mutation promotes aggregation of α-syn in vitro and exhibits PD-like pathology in cellular and animal models^34,35^. In contrast, the effect of the A30P mutation on aggregation of α-syn has been variously reported to slow down^36^, accelerate^37–39^, or not affect the aggregation rates^40^. The recent kinetic analysis of aggregation into amyloid fibrils for these α-syn variants suggested that the mutations enhance the lipid-induced primary and secondary nucleations without affecting fibril elongation^33^.

Familial PD mutations are also known to enhance the intracellular truncation of the C-terminal regions of α-syn^41,42^. The C-terminally truncated fragments of α-syn are another characteristic species enriched in the intraneuronal deposits of PD patients^41–44^. The accumulation of aggregated α-syn in lysosome leads to incomplete degradation by cathepsins, resulting in the generation of C-terminally truncated fragments (e.g. Δ104‒140, Δ120‒140, and Δ123‒140)^41–43^. These C-terminally truncated fragments have higher amyloid-forming propensity and amyloid-seeding potency than full-length α-syn^42,43,45^. In addition, it was reported that amyloid fibrils of the Δ104‒140 or Δ120‒140 fragments of α-syn are more twisted than wild-type (WT) fibrils, and the greater twisted morphologies are propagated onto WT fibrils upon cross-seeding^46^. Overexpression of C-terminally truncated forms of α-syn in *Drosophila* and in murine demonstrated that these fragments tend to form more pathologic inclusions than full-length protein, inducing dopaminergic cell death^42,47^.

In the present study, we asked how the PD-related mutations or truncations modulate aggregation and fibril formation of α-syn using the point (A30P and A53T) and C-terminally truncated (Δ123‒140 and Δ104‒140) variants (Fig. 1). A30 residue is located at the center of the N-terminal region that has no amyloidogenicity and low hydropathy, whereas A53 residue is on the highly amyloidogenic and hydrophobic pre-NAC region^48,49^, which forms interface of two protofilaments in the fibril core^50–52^. Truncations at N122 or N103 residues remove 7 or 14 acidic residues from the C-terminal domain which contains 15 negatively charged residues. Aggregation kinetics of these α-syn variants were analyzed by the Finke–Watzky 2-step model, which has been applied to separate homogeneous nucleation from heterogeneous autocatalytic fibril growth^53–55^. We also estimated the contribution of secondary processes (fibril fragmentation and surface-catalyzed secondary nucleation) to fibril growth based on the dependencies of fibril formation on the total protein concentration^56,57^. In addition, we explored the effect of temperature on the kinetics of the aggregation of α-syn variants into amyloid fibrils to uncover the thermodynamic mechanism of nucleation and fibril elongation of the α-syn variants.

## Results

### Kinetic analyses of fibril formation of α-syn variants

**Figure 2:**
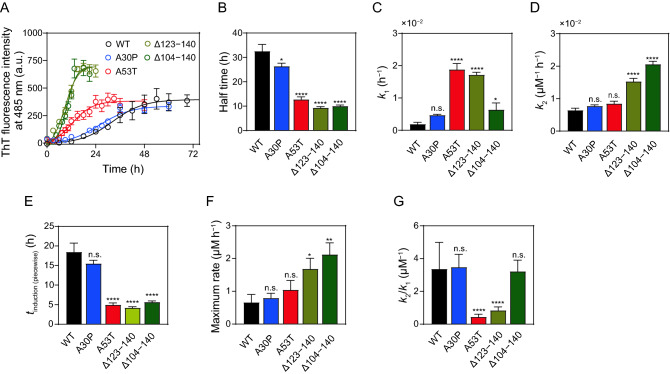
Kinetics of fibril formation of α-syn variants monitored by ThT fluorescence. (**A**) ThT fluorescence traces for α-syn fibrillization. WT (black), A30P (blue), A53T (red), Δ123–140 (yellow), and Δ104–140 (green). Solutions of monomeric α-syn variants (20 μM in PBS, pH 7.4) were incubated at 37 °C with shaking at 500 rpm. Each experiment was repeated at least triplicated. *Error bars* represent S.E. The *solid lines* are the fitted curves by the Finke–Watzky 2-step model. *a.u.*, arbitrary unit. (**B**) Comparison of half time for fibril formation of α-syn variants. Half times were obtained according to the sigmoidal Eq. (1). (**C**,**D**) Comparison of rate constants for nucleation (*k*_1_) and autocatalytic fibril growth (*k*_2_) of α-syn variants according to the Finke–Watzky Eq. (2). (**E**–**G**) Comparison of *t*_induction (piecewise)_, maximum rate, and *k*_2_/*k*_1_ ratio for α-syn variants calculated from *k*_1_ and *k*_2_ values according to Eqs. (3) and (4). *Error bars* represent S.E. *p < 0.05; **p < 0.01; ****p < 0.0001 versus WT. n.s., not significant.

We first examined the effects of PD-related mutations or C-terminal truncations on the kinetics of fibril formation using a fibril-specific fluorescent dye, thioflavin T (ThT). The time courses of increase in ThT fluorescence intensity were fitted by the empirical sigmoidal curves composed of an initial lag phase, a rapid growth phase, and a final plateau phase^58,59^ (Fig. 2A), underlying nucleation-polymerization mechanism^60,61^. Significant acceleration of increases in ThT fluorescence was observed for the A53T, Δ123‒140, and Δ104‒140 variants compared to WT. Comparison of the half times at which the ThT fluorescence reaches 50% of the plateau phase indicates that A53T, Δ123‒140, and Δ104‒140 variants have an ability to form amyloid fibrils much faster than WT, whereas the A30P mutation has a little effect (Fig. 2B). Comparison of the lag times for nucleation and apparent rate constants for fibril growth, *k*_app_ obtained by the sigmoidal equation (Supplementary Figs. S1A and S1B) indicated that C-terminal 123‒140 or 104‒140 truncations significantly enhance both nucleation and fibril growth, whereas the A53T mutation only enhances the nucleation^61^.

The kinetic curves of ThT fluorescence increase were also analyzed by the Finke–Watzky 2-step kinetic model to obtain rate constants for homogeneous nucleation (*k*_1_) and autocatalytic fibril growth (*k*_2_)^53–55^. As shown in Figs. 2C and 2D, significant increase in the rate constants for nucleation was observed for the A53T, Δ123‒140 and Δ104‒140 variants, whereas the Δ123‒140 and Δ104‒140 truncations significantly increased the rate constants for fibril growth. Based on the *k*_1_ and *k*_2_ values, we also calculated the induction period *t*_induction (piecewise)_ and the maximum rate of fibril growth^62^ which correspond to lag time for nucleation and apparent rate constant for fibril growth, respectively, in the empirical sigmoidal equation. Comparison of the induction periods and maximum rates further demonstrated that the C-terminal 123‒140 or 104‒140 truncations greatly decrease the induction period and increase the maximum rate for fibril growth, whereas the A53T mutation significantly decreases the induction period (Fig. 2E,F). We note that the linear correlations between *t*_induction (piecewise)_ and the lag time (Supplementary Fig. S1C) as well as between the maximum rate and *k*_app_ (Supplementary Fig. S1D) were observed for all variants. In addition, Fig. 2G compares *k*_2_/*k*_1_ ratio that reflects the relative dominance of nucleation and fibril growth processes. The A53T and Δ123‒140 variants exhibit much lower *k*_2_/*k*_1_ ratio compared to WT, indicating greatly promoted homogeneous nucleation than autocatalytic fibril growth. In contrast, the Δ104‒140 shows similar *k*_2_/*k*_1_ ratio to WT, suggesting the simultaneous promotion of nucleation and fibril growth. These kinetic analyses indicate that the A53T, Δ123‒140, and Δ104‒140 modifications accelerate amyloid fibril formation of α-syn possibly by different mechanisms.

### Characterization of aggregates formed by α-syn variants

**Figure 3:**
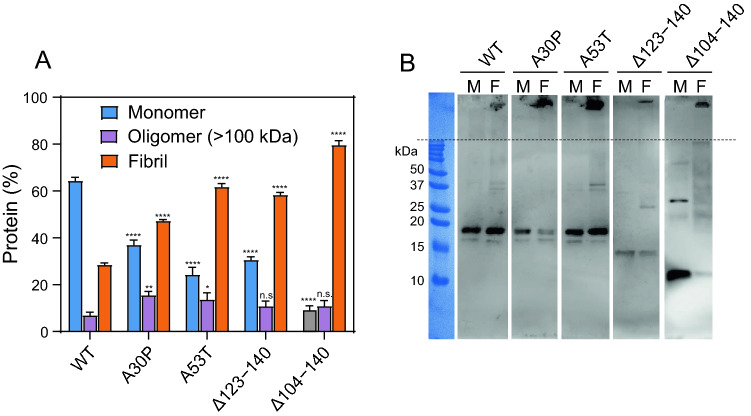
Determination of α-syn species at plateau phase of fibril formation. (**A**) Quantitative analysis of relative amounts of monomeric, oligomeric, and fibrillar forms of α-syn at plateau phase. Solution of 20 μM monomeric α-syn was incubated at 37 °C for 3 days with shaking. After the incubation, amyloid fibrils were precipitated by ultracentrifugation, and the resultant supernatant was filtrated using 100-kDa cut-off filter to separate monomeric and oligomeric forms of α-syn. Amounts of proteins in each fraction were determined by the Lowry method. *Error bars* represent S.E. *p < 0.05; **p < 0.01; ****p < 0.0001 versus each form of WT. n.s., not significant. (**B**) Western blot analysis of α-syn solution after the incubation at 37 °C for 3 days. Solution of α-syn before (M, monomeric) and after (F, fibrillar) the incubation was run on 15% SDS-PAGE gels, transferred to PVDF membrane, and detected using anti-α-syn polyclonal antibody for WT, A30P, A53T, and Δ123–140 or anti-α-syn N103 fragment polyclonal antibody for Δ104–140. The amounts of loaded protein were 250 ng/lane for WT, A30P, A53T, and Δ123–140 and 1 μg/lane for Δ104–140. The dotted line represents a border between stacking and separating gels.

We next compared the amounts of monomer, oligomer, and fibrils at plateau phase in ThT fluorescence curves for α-syn variants according to the ultracentrifugation and filtration procedure^63,64^. Figure 3A shows that in our experimental condition, about 30% of WT α-syn existed as amyloid fibrils and large amount of protein remained as monomers. We note that less than 10% of WT α-syn existed as oligomers in equilibrium at plateau phase^65^. For all α-syn variants, significant increase in the amount of amyloid fibrils and concomitant decrease in monomer were observed, especially for the Δ104‒140 variant, about 80% of α-syn exist as fibrils. Consistently, western blotting analysis showed the coexistence of monomers with oligomers and fibrils at equilibrium, and that the PD-related modifications shifted the equilibrium to oligomer and fibrillar forms (Fig. 3B). These results indicate that the PD-related modifications of α-syn promote the conversion of monomers to amyloid fibrils at equilibrium.

**Figure 4:**
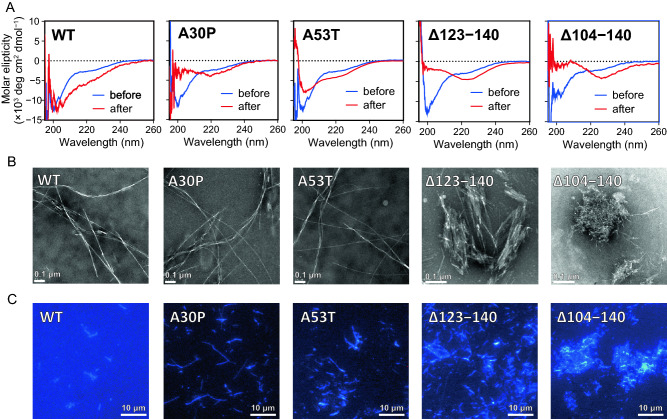
Far-UV CD spectra, TEM and TIRFM images of α-syn variants after 3-day incubation. (**A**) Far-UV CD spectra of α-syn variants before (blue line) and after (red line) the incubation at 37 °C for 3 days. Protein concentration was 20 μM. *deg*, degrees. (**B**) TEM images of α-syn variants after 3-day incubation. *Scale bar* represents 0.1 μm. (**C**) TIRFM images of α-syn variants after 3-day incubation. Amyloid fibrils were stained by ThT. Scale bar represents 10 μm.

Figure 4A shows the secondary structural changes of α-syn variants during incubation monitored by circular dichroism (CD) measurements. Before incubation, all monomeric α-syn variants displayed single negative peak below 200 nm, implying the random coil structure. After the incubation, the peak development around 220 nm reflecting transition to β-structure was detected for all α-syn variants, especially for the Δ123‒140 and Δ104‒140 variants. We also observed the morphology and structure of fibrils formed by α-syn variants using transmission electron microscopy (TEM) and total internal reflection fluorescence microscopy (TIRFM). TEM observation demonstrated that WT, A30P, and A53T variants formed long, straight fibrils, whereas the Δ123‒140 and Δ104‒140 variants formed shorter, aggregated fibrils (Fig. 4B). Such accumulation of aggregated fibrils in the Δ123‒140 and Δ104‒140 variants were also observed by TIRFM (Fig. 4C). These results indicate that the C-terminal truncation of residues 123‒140 or 104‒140 promotes the β-transition, and enhances aggregation and fibril formation of α-syn.

### Concentration dependence of aggregation kinetics of α-syn variants

**Figure 5:**
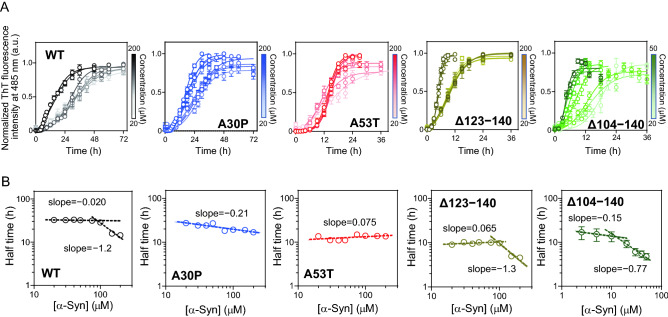
Dependency of fibril formation kinetics on initial monomer concentration of α-syn variants. (**A**) Normalized ThT fluorescence curves for fibrilization of α-syn at varying initial monomer concentration. Each experiment was repeated at least triplicated. *Error bars* represent S.E. The *solid lines* are the fitted curves by the Finke–Watzky 2-step model. (**B**) Double logarithmic plots of the half time for fibril formation and initial monomer concentration. Half time was determined according to the sigmoidal Eq. (1). The *dashed lines* are the linear regression lines. Values of the slopes were also shown. *Error bars* represent S.E.

To understand the aggregation mechanism of the α-syn variants, we investigated the dependencies of kinetic parameters of amyloid fibril formation on the monomer concentration of α-syn. Figure 5A shows the normalized time courses of ThT fluorescence for α-syn variants at varying monomer concentrations. The dependence of the half time on the initial monomer concentration provides an indication of the dominant step in the aggregation process^57,66^. Figure 5B shows double logarithmic plots of the half time and initial monomer concentration for α-syn variants. The slopes of the plots for all α-syn variants were close to 0 at relatively low concentrations, reflecting a weak concentration dependence of the half time. This indicates that structural conversion of monomer at fibril ends (known as saturated elongation) is a dominant process for fibrilization of α-syn at low protein concentration^66^. At high monomer concentrations, however, the slope of the plots became steeper for WT, Δ123‒140, and Δ104‒140 variants, at which inflection concentration was around 100 μM for WT and the Δ123‒140, and 10 μM for the Δ104‒140, respectively. This indicates that the microscopic mechanism of fibril formation changes over these concentration ranges such that competition of processes such as secondary nucleation is present in parallel, in which formation of amyloid nuclei is catalyzed on the surfaces of existing fibrils^56,67–69^. Indeed, the concomitant reduction of *t*_induction (piecewise)_ and increase in maximum rate (Supplementary Fig. S3) imply the acceleration of both nucleation and fibril growth by secondary nucleation pathway^70^. In contrast, linear correlations in the concentration ranges of 20–200 µM were seen for the A30P and A53T variants, indicating that the dominant mechanism does not change in these concentration range. These results suggest that at physiological concentration of α-syn (around 20 µM), the Δ104‒140 truncation enhances not only the saturated elongation process but also the autocatalytic secondary nucleation, leading to great acceleration of amyloid fibril formation by α-syn.

### Seeded aggregation assay of α-syn variants

**Figure 6:**
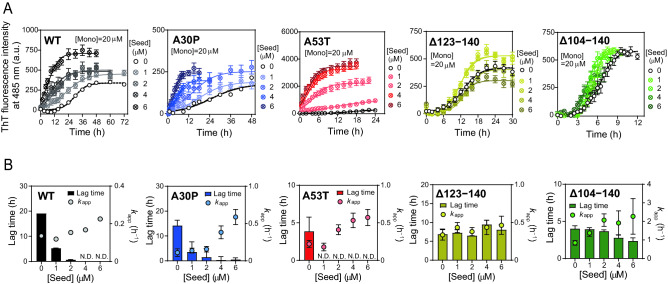
Seeded aggregation assay for α-syn variants with varying concentration of seed fibrils. (**A**) Traces of ThT fluorescence for fibril formation of α-syn with varying concentration of seed fibrils. Each experiment was repeated at least triplicated. *Error bars* represent S.E. The *solid* (seeded) and *dashed* (unseeded) *lines* are the fitted curves by the Finke–Watzky 2-step model. *a.u.*, arbitrary unit. (**B**) Comparison of lag time and apparent rate constant for fibril growth *k*_app_ in fibril formation of α-syn variants with varying concentration of seed fibrils. *Error bars* represent S.E. *N.D.*, not determined.

To obtain more detailed information about fibril formation mechanism of α-syn variants, seeded aggregation assays were performed. Figure 6A shows ThT fluorescence curves for 20 μM of monomeric α-syn variants in the presence of varying concentration of preformed seed fibrils. Effect of seed concentration on the lag times and *k*_app_ values based on the fitting by the sigmoidal equation were shown in Fig. 6B. Increase in seed concentration gradually reduced the lag time and increased *k*_app_ for WT, A30P, and A53T variants. Such dependency of seeding efficiency on the relative concentration of seed fibrils to monomers agrees with the nucleation-polymerization model at equilibrium between monomer and nuclei^71,72^. In contrast, the lag time for the Δ123‒140 and Δ104‒140 variants did not change even in the presence of 6 μM of seed fibrils that corresponds to 30% of monomer concentration (Fig. 6B). This indicates that the amount of seed fibrils is not a dominant factor for the kinetics of fibril formation by the C-terminally truncated variants.

### Thermodynamic analysis of fibril formation of α-syn variants

**Figure 7:**
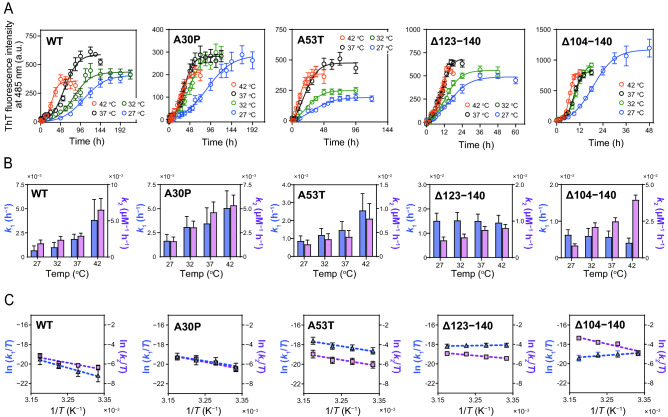
Temperature dependence of fibril formation kinetics of α-syn variants. (**A**) ThT fluorescence curves for fibril formation of α-syn at varying temperature. Each experiment was repeated at least triplicated. *Error bars* represent S.E. (**B**) Temperature dependencies of rate constants for nucleation (*k*_1_, light blue) and fibril growth (*k*_2_, light purple) for fibril formation of α-syn variants. The rate constants were determined according to the Finke–Watzky 2-step model. *Error bars* represent S.E. (**C**) Eyring plots of rate constants of *k*_1_ and *k*_2_ for fibril formation. *Error bars* represent S.E. The dashed lines represent linear regression lines.

To further gain thermodynamic characteristics of fibril formation by the α-syn variants, we explored the effects of temperature on the fibril formation kinetics. Figure 7A shows the time courses of ThT fluorescence for α-syn variants at different temperatures. Fibril formation of all variants was accelerated with increasing temperature and accordingly, temperature-dependent decreases in the half times for fibril formation were observed (Supplementary Fig. S4). As shown in Fig. 7B, the rate constants for nucleation (*k*_1_) and fibril growth (*k*_2_) obtained from the Finke–Watzky 2-step model analysis increased with temperature except for *k*_1_ values of the Δ123‒140 and Δ104‒140 variants, in which *k*_1_ values decreased with increasing temperature. This suggests that the thermodynamic pathway for nucleation of the Δ123‒140 and Δ104‒140 variants is different from that for other α-syn variants.

Eyring plots for the rate constants of *k*_1_ and *k*_2_ (Fig. 7C) gave the thermodynamic parameters for nucleation and fibril growth, and the obtained activation enthalpy Δ*H*^‡^, activation entropy Δ*S*^‡^, and activation Gibbs free energy Δ*G*^‡^ for nucleation and fibril growth are listed in Table 1. Consistent with the previous report^54^, both nucleation and fibril growth of WT α-syn were enthalpically and entropically unfavorable, and the free energy barrier mainly consists of the enthalpic barrier. The A30P and A53T mutations significantly decreased the enthalpic barrier for nucleation with concomitant increase in the entropic barrier. Significantly, the activation enthalpy for nucleation was reversed in sign by the C-terminal 123‒140 or 104‒140 truncations with compensative increase in the entropic barrier, indicating that the free energy barrier for nucleation of the Δ123‒140 and Δ104‒140 variants are entirely entropic. For fibril growth, the thermodynamic parameters of the A30P and A53T variants are similar to those of WT. A large reduction in the unfavorable activation enthalpy was observed for the Δ123‒140, whereas reverse in sign occurred in the activation entropy of the Δ104‒140 variant so that the fibril growth is entropically favorable. Thus, these results indicate that the C-terminal truncations of the Δ123‒140 or Δ104‒140 have crucial effects on the thermodynamic properties of nucleation and fibril growth in amyloid fibril formation by α-syn.

**Table 1:** Thermodynamic parameters for nucleation and fibril growth of fibril formation of α-syn variants.

	Nucleation, *k*_1_			Fibril growth, *k*_2_		
	Δ*H*^‡^ (kJ mol^−1^)	*T*Δ*S*^‡^ at 37 °C (kJ mol^−1^)	Δ*G*^‡^ at 37 °C (kJ mol^−1^)	Δ*H*^‡^ (kJ mol^−1^)	*T*Δ*S*^‡^ at 37 °C (kJ mol^−1^)	Δ *G*^‡^ at 37 °C (kJ mol^−1^)
WT	87 ± 9	− 26 ± 9	113 ± 1	59 ± 16	− 17 ± 16	77 ± 1
A30P	52 ± 10*	− 60 ± 10*	112 ± 1	61 ± 12	− 15 ± 11	75 ± 1
A53T	52 ± 8*	− 56 ± 8*	108 ± 1***	53 ± 12	− 22 ± 11	75 ± 1
Δ123–140	− 5 ± 2****	− 113 ± 2****	108 ± 1**	28 ± 5	− 47 ± 5	74 ± 1
Δ104–140	− 23 ± 8****	− 134 ± 8****	110 ± 1	73 ± 16	2 ± 16	71 ± 1****

The values of Δ*H*^‡^ and Δ*S*^‡^ were determined from the slope and y-intercept of Eyring plots shown in Fig. 7C. The values of Δ*G*^‡^ were calculated from *k*_1_ and *k*_2_ values at 37 °C according to Eq. (6). *p < 0.05; **p < 0.01; ***p < 0.0005; ****p < 0.0001 versus each parameter of WT.

## Discussion

Among familial PD mutations, the A30P and A53T are frequent mutations in α-syn inducing early-onset familial PD^25–27^. The C-terminally Δ123‒140 or Δ104‒140 truncated fragments of α-syn are enriched in deposits in PD brains, having higher neurotoxicity than full-length form^41–44^. In the present study, we used biophysical approaches to understand the kinetics and equilibrium of amyloid fibril formation by these α-syn variants. We demonstrated that these PD-related modifications in α-syn shift the equilibrium from monomeric to fibrillar forms (Fig. 3). In contrast, the effects of these modifications on the fibril formation kinetics are different: the A53T, Δ123‒140, and Δ104‒140 modifications significantly accelerated the fibril formation, whereas the A30P mutation did not (Fig. 2A,B). Kinetic analyses using the Finke–Watzky 2-step model further revealed that the A53T mutation promotes homogeneous nucleation, whereas the C-terminal 123‒140 or 104‒140 truncations accelerate both nucleation and fibril growth (Fig. 2C,D).

In addition to the kinetic analyses, we performed thermodynamic analyses of ThT fluorescence kinetics to gain further insights into the molecular mechanism of aggregation and fibril formation of the α-syn variants. Consistent with the previous report^54^, the nucleation and fibril growth of WT α-syn were shown to be both enthalpically and entropically unfavorable, in which the energy barriers are mainly enthalpic (Fig. 7 and Table 1). We also demonstrated that both the A30P and A53T mutations decrease this enthalpic barrier for nucleation without affecting the thermodynamic parameters for fibril growth. Given that the electrostatic intramolecular interactions between the N- and C-terminal regions in the monomeric α-syn are thought to suppress amyloid fibril formation by preventing solvent-exposure of the aggregation-prone NAC region^73–75^, it is plausible that the enthalpic barrier of nucleation comes partly from breakage of the intramolecular interactions in the monomeric α-syn. Thus, it is likely that the A53T mutation located at the aggregation-prone pre-NAC region (Fig. 1B)^48,49^ alters the intramolecular interactions between the N- and C-terminal regions^74,76,77^, resulting in the decreased enthalpic barrier for nucleation. To support this idea, the previous NMR studies suggested that PD-related mutations destabilize the long-range interactions between the mutated site and C-terminal residues^76,77^, resulting in the formation of aggregation-prone intermediates with exposure of highly amyloidogenic region (residues 54‒60)^76^. Regarding the fibril growth process, the unfavorable activation entropy of α-syn is thought to arise because resolvation of the fibril surface dominates over desolvation of the free monomer^69,78^. Large elimination of negatively charged residues by the C-terminal 104‒140 truncation may alter the balance of resolvation of the fibril surface and desolvation of the monomer, resulting in the favorable activation entropy observed for the Δ104‒140 variant (Table 1).

It is known that monomer-dependent secondary nucleation on the surface of already existing fibrils plays a dominant role in fibril formation of α-syn^68,79^. Quantitative analysis of α-syn aggregates indicated that compared to WT α-syn, the A30P and A53T mutations greatly increase the amount of oligomers at plateau phase (Fig. 3A)^39,40,80–83^. This may explain why the secondary nucleation of the A30P and A53T variants was suppressed even at high protein concentration (≥ 100 μM) (Fig. 5). The decrease in the enthalpic barrier of nucleation for the A30P and A53T variants would enhance the oligomerization of α-syn, likely decreasing the amount of free monomer required for secondary nucleation at the growth phase. Interestingly, such enthalpic effect on the nucleation of the α-syn variants is in sharp contrast to the case of apolipoprotein A-I: the nucleation process of the G26R variant of the N-terminal 1‒83 fragment of apolipoprotein A-I is enthalpically unfavorable but entropically favorable^78^. In this case, the favorable activation entropy of nucleation is thought to come from desolvation of highly amyloidogenic residues^67,84^.

A significant finding in this study is that the C-terminal 123‒140 or 104‒140 truncations in α-syn cause the nucleation process to be enthalpically favorable, with the free energy barriers being entirely entropic (Fig. 7 and Table 1). Such enthalpically favorable nucleation is consistent with the feature of secondary nucleation^73,74^, in which the nucleation-competent conformation of monomer is enthalpically stabilized by adsorption onto fibril surfaces. Indeed, analyses of the dependence of kinetic parameter on the monomer concentration (Fig. 5) indicated the markedly enhanced secondary nucleation for the Δ104‒140 variant. However, the truncation of residues 123‒140 did not enhance the secondary nucleation compared to WT. Direct observation of secondary nucleation revealed that secondary nucleation is a multistep process involving the attachment of monomers to fibril surfaces, followed by structural conversion and detachment of daughter aggregates^85^. Previous simulation study indicated that too strong adsorption of proteins onto fibril surfaces rather hampers the secondary nucleation by suppressing dissociation of newly formed nuclei from fibril surfaces. Thus, it is possible that the secondary nucleation occurs only at the optimal monomer-fibril interactions^86^. During the peer review period, the most recent study has reported that the C-terminal truncated α-syn variants with shorter than 125 residues exhibit the markedly enhanced secondary process of fibril formation, most likely fragmentation^87^. We note that in the secondary processes of fibril formation, it is very difficult to distinguish the saturated secondary nucleation from the fragmentation^57,66^.

It was reported that electrostatic interactions of positively charged N-terminal residues (K6, K10, and K12) of monomeric α-syn with negatively charged C-terminal ends of fibrils^52,88^ are involved in adsorption of monomers onto fibril surfaces^89^. In contrast, the electrostatic repulsion between monomers as well as monomers and fibrils at the C-terminal regions is thought to suppress the secondary nucleation^45,69,79^. Thus, it is plausible that the truncations in the C-terminal region alter the dominance of secondary nucleation. In this regard, our kinetic and thermodynamic analyses might indicate that the adsorption of nuclei onto fibril surfaces in the Δ104–140 is optimal to induce secondary nucleation, whereas that for the Δ123–140 is too strong due to the additional interactions in the remained C-terminus (Fig. 1A). In this scenario, the entropically favorable fibril growth of the Δ104–140 may reflect the dissociation of nuclei from fibril surfaces during fibril growth, whereas the enthalpically and entropically unfavorable fibril growth of the Δ123–140 as well as WT α-syn implies the fibril elongation without the dissociation of nuclei (Table 1)^66,68,85^.

**Figure 8:**
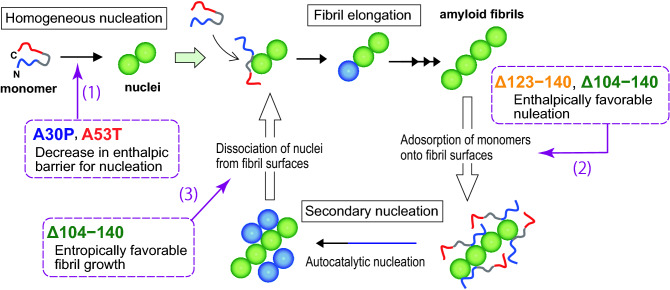
Schematic representation of the effects of the PD-related mutations or C-terminal truncations on the fibril-forming process of α-syn. Fibril formation of α-syn occurs through homogeneous nucleation and fibril elongation, whereas secondary nucleation process contributes to the autocatalytic enhancement of fibril formation at high initial monomer concentration (≥ 100 μM). Both the A30P and A53T mutations enhance the homogeneous nucleation by decreasing the enthalpic barrier for nucleation (1). The enthalpically favorable nucleation of the Δ123–140 and Δ104–140 variants suggests the enhanced adsorption of monomers onto fibril surfaces (2). The entropically favorable fibril growth of the Δ104–140 variant reflects the dissociation of nuclei from fibril surfaces, followed by the fibril elongation (3). See the text for more detail.

In summary, the present study provides for the first time the thermodynamic basis for the enhanced aggregation and fibril formation of the PD-related α-syn variants as summarized in Fig. 8. The N-terminal A30P and A53T mutations enhance the oligomerization of α-syn through the decreased enthalpic barrier for nucleation. In the C-terminally truncated Δ123‒140 and Δ104‒140 variants, the nucleation process is enthalpically favorable with the free energy barrier being entirely entropic, thereby promoting interaction of monomers with fibril surfaces in the autocatalytic fibril formation. In physiological neurons, the PD-related C-terminally truncated α-syn fragments are formed through an incomplete degradation of amyloid fibrils by cathepsins^41–43^, and the resulted aggregates can spread between neighboring cells in a prion-like manner^90–92^. Thus, our findings will provide novel molecular basis to understand the relationships between the aggregation and propagation properties of α-syn variants and PD pathogenesis.

## Materials and methods

### Preparation of recombinant human α-syn proteins

Recombinant human α-syn and its PD-related variants (A30P, A53T, Δ123‒140, and Δ104‒140) were expressed in *E. coli* BL21 Star (DE3) with N-terminal thioredoxin and hexa-histidine tags, and isolated by Ni-affinity chromatography as previously reported^93^. The fused tags were cleaved with HRV-3C protease, and then removed using Ni-chelating resin column. The obtained α-syn proteins have two extra amino acids, Gly-Pro at the N-terminus. The purity of proteins was estimated as at least 95% by Coomassie-stained SDS-PAGE.

### ThT fluorescence assay

The lyophilized α-syn proteins were dissolved in 20 mM glycine buffer (pH 8.0) and solubilized by addition of 2 M NaOH^94,95^. The solution was centrifuged at 10,000*g* for 30 min at 4 °C to remove insoluble aggregates, and then dialyzed against PBS containing 0.02% NaN_3_ overnight. After dialysis, the solution was again centrifuged at 10,000*g* for 30 min at 4 °C and the supernatant was used in the experiment. The protein concentration was determined by the Lowry method. The monomer solution supplemented with 10 μM ThT was pipetted into multiple wells of 96-well black plate (ThermoFisher Scientific, MA, USA) The plate was incubated at 37 °C with shaking at 500 rpm in the presence of a Teflon polybead (1/8″ diameter, ASONE, Osaka, Japan) in each well. ThT fluorescence intensity at 485 nm with an excitation wavelength of 440 nm was measured using Infinite M200 microplate reader (Tecan, Männedorf, Switzerland) at each time points.

### Preparation of α-synuclein seed fibrils

For preparing seed fibrils, 200 μM of monomeric α-syn solution was incubated in 96 well plate at 37 °C, with shaking at 500 rpm in the presence of Teflon polybeads. The formed fibrils were precipitated by ultracentrifugation in an Optima MAX-TL (Beckman Coulter, Fullerton, CA) with a TLA-120.2 rotor at 80,000 rpm for 1.5 h at 15 °C. After the centrifugation, the supernatant was carefully removed, and the pellet was washed twice with PBS. The resultant pellet of amyloid fibrils was resuspended in PBS by sonicating in the bath-type ultrasonicator (Branson 1510, Danbury, CT) for 1 min. The collected amyloid fibrils were stored at 4 ºC, and re-sonicated for 1 min before use.

### Kinetic and thermodynamic analyses of fibril formation

ThT fluorescence data for the fibril formation of α-syn were analyzed using the sigmoidal Eq.^58,96^,

1 $$ F = F_{0} + \frac{{F_{\max } - F_{0} }}{{1 + \exp \left[ {k_{app} (t_{{{1 \mathord{\left/ {\vphantom {1 2}} \right. \kern-\nulldelimiterspace} 2}}} - t)} \right]}} $$

where *F* is the fluorescence intensity, *F*_0_ is the initial baseline at the lag phase, and *F*_max_ is the final baseline at the plateau phase. *k*_app_ is the apparent rate constant for fibril growth. *t*_1/2_ is the half time for fibril formation at which the ThT fluorescence intensity reaches 50% of the intensity at plateau phase. The lag time for nucleation is calculated as *t*_1/2_ − 2/*k*_app_.

The ThT fluorescence data were also analyzed according to the Finke–Watzky 2-step nucleation-autocatalytic growth model^53,54,78^,

2 $$ \frac{{F - F_{0} }}{{F_{\max } - F_{0} }} = 1 - \frac{{k_{1} + k_{2} [{\text{A}}]_{0} }}{{k_{1} \exp \left( {k_{1} + k_{2} [{\text{A}}]_{0} } \right)t + k_{2} [{\text{A}}]_{0} }} $$

where [A]_0_ is initial concentration of monomeric protein, *k*_1_ and *k*_2_ are the rate constants for the nucleation and autocatalytic fibril growth, respectively. The kinetic parameters *t*_induction (piecewise)_ and maximum rate *v*_max_ were calculated using following Eqs.^62^,

3 $$ t_{{\text{induction(piecewise)}}} = \frac{{k_{1} + k_{2} [{\text{A}}]_{0} }}{{\left( {k_{1} - k_{2} [{\text{A}}]_{0} } \right)^{2} }}\ln \left( {\frac{{k_{2} [{\text{A}}]_{0} }}{{k_{1} }}} \right) + \frac{2}{{k_{1} - k_{2} [{\text{A}}]_{0} }} $$

4 $$ {\text{maximum rate}},v_{\max } = \frac{{\left( {k_{1} + k_{2} [A]_{0} } \right)^{2} }}{{4k_{2} }} $$

Thermodynamic parameters for nucleation and fibril growth phases were determined using the Eyring Eq. ^78^,

5 $$ \ln \left( \frac{k}{T} \right) = - \frac{{\Delta H^{\ddag } }}{R}\frac{1}{T} + \frac{{\Delta S^{\ddag } }}{R} + \ln \left( {\frac{{k_{B} }}{h}} \right) $$

6 $$ \Delta G^{\ddag } = - RT\left[ {\ln (k/T) + \ln (h/k_{B} )} \right] $$

where *k*_B_ is the Boltzmann constant and *h* is the Planck constant. The activation enthalpy (Δ*H*^‡^) and entropy (Δ*S*^‡^) were obtained from the slope and *y*-intercept of the linear fitting based on Eq. (5). Δ*G*^‡^ was calculated from *k*_1_ and *k*_2_ values at 37 °C according to Eq. (6).

### Quantification of oligomeric and fibrillar species

Separation and quantification of oligomers and fibrils were performed according to the previously reported protocol^63,64^. 20 μM of monomeric α-syn solution was incubated in 96 well plate at 37 °C with shaking at 500 rpm in the presence of Teflon polybeads for 3 days. The formed fibrils were precipitated by centrifugation in an Optima MAX-TL with a TLA120.2 rotor at 80,000 rpm for 1.5 h at 15 °C. After the centrifugation, the supernatant and pellet were collected separately. The supernatant was then filtered through an Amicon Ultra-0.5 100 kDa cut-off filter (Millipore, Billerica, MA) at 10,000*g* for 30 min at 15 °C. The flow-through fraction in filtration was regarded to contain only monomeric α-syn. After overnight incubation with 4 M urea, the protein concentration of each fraction was determined by the Lowry method. The amount of oligomer (> 100 kDa) was determined by deducting the protein concentration in flow-through fraction in filtration from that of supernatant in ultracentrifugation. The amount of fibrillar forms of α-syn was calculated by deducting the protein concentration in the supernatant fraction of ultracentrifugation from that of pellet fraction.

### Western blotting analysis

Proteins were subjected to SDS-PAGE with 15% gels and were transferred to polyvinylidene difluoride membranes (ATTO Corporation, Tokyo, Japan). After the blocking of membranes with 4% Block-Ace (KAC, Kyoto, Japan), the membranes were probed with the anti-α-syn polyclonal antibody (Proteintech, #10842-1-AP) for WT, A30P, A53T, and Δ123‒140, or with anti-α-syn N103 fragment polyclonal antibody (Millipore, #ABN2260) for the Δ104‒140, followed by horseradish peroxidase (HRP)-conjugated AffiniPure goat anti-rabbit IgG antibody (Jackson ImmunoResearch, Inc., West Grove, PA) and ECL Prime Western Blotting Detection Reagent (GE Healthcare, Milwaukee, WI).

### CD spectroscopy

Far-UV CD spectra were recorded from 190 to 260 nm at 25 °C using a Jasco J-1500 spectropolarimeter (JASCO, Tokyo, Japan). The 20 μM of α-syn solutions in PBS before and after fibrilization were subjected to the measurements in a 1 mm quartz cuvette, and the results were corrected by subtracting the buffer base line.

### TEM

TEM measurements were performed on a JEOL JEM-1200EX transmission microscope (JEOL, Tokyo, Japan) with an acceleration voltage of 80 kV. The sample was negatively stained with a phosphomolybdic acid solution for TEM observation.

### TIRFM

TIRFM measurements were performed on a fluorescence microscopic system based on an inverted microscope (IX 70, Olympus). ThT was excited using an argon laser, and the fluorescent image was filtered with a bandpass filter and visualized using an image intensifier coupled with a SIT camera^78^.

### Statistical analysis

Data were analyzed via one-way analysis of variance followed by Dunnett' multiple comparisons test by means of Prism 8 software (GraphPad Software, La Jolla, CA). Results were regarded as significant for *p* < 0.05.

## Supplementary Information

Supplementary Information.

## Data Availability

All data generated or analyzed during this study are included in this article and its Supplementary Information files.

## References

[1] Araki K (2019). Parkinson's disease is a type of amyloidosis featuring accumulation of amyloid fibrils of alpha-synuclein. Proc. Natl. Acad. Sci. U.S.A..

[2] Stefanis L (2012). Alpha-synuclein in Parkinson's disease. Cold Spring Harb. Perspect. Med..

[3] Sulzer D, Edwards RH (2019). The physiological role of alpha-synuclein and its relationship to Parkinson's Disease. J. Neurochem..

[4] Wang C (2016). Versatile structures of alpha-synuclein. Front. Mol. Neurosci..

[5] Lashuel HA, Overk CR, Oueslati A, Masliah E (2013). The many faces of alpha-synuclein: From structure and toxicity to therapeutic target. Nat. Rev. Neurosci..

[6] Meade RM, Fairlie DP, Mason JM (2019). Alpha-synuclein structure and Parkinson's disease—lessons and emerging principles. Mol. Neurodegen..

[7] Wietek J, Haralampiev I, Amoussouvi A, Herrmann A, Stockl M (2013). Membrane bound alpha-synuclein is fully embedded in the lipid bilayer while segments with higher flexibility remain. FEBS Lett..

[8] Burmann BM (2020). Regulation of α-synuclein by chaperones in mammalian cells. Nature.

[9] Alam P, Bousset L, Melki R, Otzen DE (2019). alpha-synuclein oligomers and fibrils: A spectrum of species, a spectrum of toxicities. J. Neurochem..

[10] Ghosh D, Mehra S, Sahay S, Singh PK, Maji SK (2017). alpha-synuclein aggregation and its modulation. Int. J. Biol. Macromol..

[11] Froula JM (2019). Defining alpha-synuclein species responsible for Parkinson's disease phenotypes in mice. J. Biol. Chem..

[12] Shahmoradian SH (2019). Lewy pathology in Parkinson's disease consists of crowded organelles and lipid membranes. Nat. Neurosci..

[13] Karpowicz RJ, Trojanowski JQ, Lee VM (2019). Transmission of alpha-synuclein seeds in neurodegenerative disease: Recent developments. Lab Invest..

[14] Killinger BA, Melki R, Brundin P, Kordower JH (2019). Endogenous alpha-synuclein monomers, oligomers and resulting pathology: Let's talk about the lipids in the room. NPJ Parkinsons Dis..

[15] Ingelsson M (2016). Alpha-synuclein oligomers—neurotoxic molecules in Parkinson's disease and other lewy body disorders. Front. Neurosci..

[16] Xu L, Pu J (2016). Alpha-synuclein in Parkinson's disease: From pathogenetic dysfunction to potential clinical application. Parkinsons Dis..

[17] Wong YC, Krainc D (2017). alpha-synuclein toxicity in neurodegeneration: Mechanism and therapeutic strategies. Nat. Med..

[18] Sorrentino ZA, Giasson BI, Chakrabarty P (2019). α-Synuclein and astrocytes: Tracing the pathways from homeostasis to neurodegeneration in Lewy body disease. Acta Neuropathol..

[19] Sorrentino ZA, Giasson BI (2020). The emerging role of alpha-synuclein truncation in aggregation and disease. J. Biol. Chem..

[20] Valera E, Masliah E (2016). Therapeutic approaches in Parkinson's disease and related disorders. J. Neurochem..

[21] Brundin P, Dave KD, Kordower JH (2017). Therapeutic approaches to target alpha-synuclein pathology. Exp. Neurol..

[22] Arosio P, Vendruscolo M, Dobson CM, Knowles TP (2014). Chemical kinetics for drug discovery to combat protein aggregation diseases. Trends Pharmacol. Sci..

[23] Giorgetti S, Greco C, Tortora P, Aprile FA (2018). Targeting amyloid aggregation: An overview of strategies and mechanisms. Int. J. Mol. Sci..

[24] Chia S (2018). SAR by kinetics for drug discovery in protein misfolding diseases. Proc. Natl. Acad. Sci. U.S.A..

[25] Fujioka S (2014). Update on novel familial forms of Parkinson's disease and multiple system atrophy. Parkinsonism Relat. Disord..

[26] Polymeropoulos MH (1997). Mutation in the alpha-synuclein gene identified in families with Parkinson's disease. Science.

[27] Krüger R (1998). Ala30Pro mutation in the gene encoding alpha-synuclein in Parkinson's disease. Nat. Genet..

[28] Zarranz JJ (2004). The new mutation, E46K, of alpha-synuclein causes Parkinson and Lewy body dementia. Ann. Neurol..

[29] Appel-Cresswell S (2013). Alpha-synuclein p.H50Q, a novel pathogenic mutation for Parkinson's disease. Mov. Disord..

[30] Hoffman-Zacharska D (2013). Novel A18T and pA29S substitutions in α-synuclein may be associated with sporadic Parkinson's disease. Parkinsonism Relat. Disord..

[31] Kiely AP (2013). α-Synucleinopathy associated with G51D SNCA mutation: A link between Parkinson's disease and multiple system atrophy?. Acta Neuropathol..

[32] Pasanen P (2014). Novel α-synuclein mutation A53E associated with atypical multiple system atrophy and Parkinson's disease–type pathology. Neurobiol. Aging.

[33] Flagmeier P (2016). Mutations associated with familial Parkinson's disease alter the initiation and amplification steps of alpha-synuclein aggregation. Proc. Natl. Acad. Sci. U.S.A..

[34] Narhi L (1999). Both familial Parkinson's disease mutations accelerate alpha-synuclein aggregation. J. Biol. Chem..

[35] Giasson BI (2002). Neuronal alpha-synucleinopathy with severe movement disorder in mice expressing A53T human alpha-synuclein. Neuron.

[36] Lemkau LR (2012). Mutant protein A30P α-synuclein adopts wild-type fibril structure, despite slower fibrillation kinetics. J. Biol. Chem..

[37] Li J, Uversky VN, Fink AL (2002). Conformational behavior of human alpha-synuclein is modulated by familial Parkinson's disease point mutations A30P and A53T. Neurotoxicology.

[38] Li J, Uversky VN, Fink AL (2001). Effect of familial Parkinson's disease point mutations A30P and A53T on the structural properties, aggregation, and fibrillation of human alpha-synuclein. Biochemistry.

[39] Lashuel HA (2002). Alpha-synuclein, especially the Parkinson's disease–associated mutants, forms pore-like annular and tubular protofibrils. J. Mol. Biol..

[40] Conway KA (2000). Acceleration of oligomerization, not fibrillization, is a shared property of both alpha-synuclein mutations linked to early-onset Parkinson's disease: Implications for pathogenesis and therapy. Proc. Natl. Acad. Sci. U.S.A..

[41] Li W (2005). Aggregation promoting C-terminal truncation of alpha-synuclein is a normal cellular process and is enhanced by the familial Parkinson's disease–linked mutations. Proc. Natl. Acad. Sci. U.S.A..

[42] Zhang Z (2017). Asparagine endopeptidase cleaves alpha-synuclein and mediates pathologic activities in Parkinson's disease. Nat. Struct. Mol. Biol..

[43] McGlinchey RP (2019). C-terminal alpha-synuclein truncations are linked to cysteine cathepsin activity in Parkinson's disease. J. Biol. Chem..

[44] Baba M (1998). Aggregation of alpha-synuclein in Lewy bodies of sporadic Parkinson's disease and dementia with Lewy bodies. Am. J. Pathol..

[45] van der Wateren IM, Knowles TPJ, Buell AK, Dobson CM, Galvagnion C (2018). C-terminal truncation of alpha-synuclein promotes amyloid fibril amplification at physiological pH. Chem. Sci..

[46] Ni X, McGlinchey RP, Jiang J, Lee JC (2019). Structural insights into α-synuclein fibril polymorphism: Effects of Parkinson's disease-related C-terminal truncations. J. Mol. Biol..

[47] Periquet M, Fulga T, Myllykangas L, Schlossmacher MG, Feany MB (2007). Aggregated alpha-synuclein mediates dopaminergic neurotoxicity in vivo. J. Neurosci..

[48] Rodriguez JA (2015). Structure of the toxic core of α-synuclein from invisible crystals. Nature.

[49] Doherty CPA (2020). A short motif in the N-terminal region of alpha-synuclein is critical for both aggregation and function. Nat. Struct. Mol. Biol..

[50] Li Y (2018). Amyloid fibril structure of α-synuclein determined by cryo-electron microscopy. Cell Res..

[51] Li B (2018). Cryo-EM of full-length α-synuclein reveals fibril polymorphs with a common structural kernel. Nat. Commun..

[52] Guerrero-Ferreira R (2018). Cryo-EM structure of alpha-synuclein fibrils. Elife.

[53] Morris AM, Watzky MA, Finke RG (2009). Protein aggregation kinetics, mechanism, and curve-fitting: A review of the literature. Biochim. Biophys. Acta.

[54] Morris AM, Finke RG (2009). Alpha-synuclein aggregation variable temperature and variable pH kinetic data: A re-analysis using the Finke-Watzky 2-step model of nucleation and autocatalytic growth. Biophys. Chem..

[55] Morris AM, Watzky MA, Agar JN, Finke RG (2008). Fitting neurological protein aggregation kinetic data via a 2-step, minimal/"Ockham's razor" model: The Finke-Watzky mechanism of nucleation followed by autocatalytic surface growth. Biochemistry.

[56] Cohen SI (2013). Proliferation of amyloid-beta42 aggregates occurs through a secondary nucleation mechanism. Proc. Natl. Acad. Sci. U.S.A..

[57] Meisl G (2016). Molecular mechanisms of protein aggregation from global fitting of kinetic models. Nat. Protoc..

[58] Gade Malmos K (2017). ThT 101: A primer on the use of thioflavin T to investigate amyloid formation. Amyloid.

[59] Holubová M (2021). Does polysaccharide glycogen behave as a promoter of amyloid fibril formation at physiologically relevant concentrations?. Soft Matter.

[60] Wordehoff MM, Hoyer W (2018). Alpha-synuclein aggregation monitored by thioflavin T fluorescence assay. Bio Protoc..

[61] Shoffner SK, Schnell S (2016). Estimation of the lag time in a subsequent monomer addition model for fibril elongation. Phys. Chem. Chem. Phys..

[62] Bentea L, Watzky MA, Finke RG (2017). Sigmoidal nucleation and growth curves across nature fit by the finke-watzky model of slow continuous nucleation and autocatalytic growth: Explicit formulas for the lag and growth times plus other key insights. J. Phys. Chem. C.

[63] Ray S (2020). alpha-Synuclein aggregation nucleates through liquid–liquid phase separation. Nat. Chem..

[64] Kumar ST (2020). A simple, versatile and robust centrifugation-based filtration protocol for the isolation and quantification of α-synuclein monomers, oligomers and fibrils: Towards improving experimental reproducibility in α-synuclein research. J. Neurochem..

[65] Zurlo E (2021). In situ kinetic measurements of alpha-synuclein aggregation reveal large population of short-lived oligomers. PLoS ONE.

[66] Meisl G (2017). Scaling behaviour and rate-determining steps in filamentous self-assembly. Chem. Sci..

[67] Cohen SIA (2018). Distinct thermodynamic signatures of oligomer generation in the aggregation of the amyloid-beta peptide. Nat. Chem..

[68] Tornquist M (2018). Secondary nucleation in amyloid formation. Chem. Commun. (Camb).

[69] Gaspar R (2017). Secondary nucleation of monomers on fibril surface dominates alpha-synuclein aggregation and provides autocatalytic amyloid amplification. Q. Rev. Biophys..

[70] Arosio P, Knowles TP, Linse S (2015). On the lag phase in amyloid fibril formation. Phys. Chem. Chem. Phys..

[71] Hoshino M (2017). Fibril formation from the amyloid-β peptide is governed by a dynamic equilibrium involving association and dissociation of the monomer. Biophys. Rev..

[72] Bishop MF, Ferrone FA (1984). Kinetics of nucleation-controlled polymerization. A perturbation treatment for use with a secondary pathway. Biophys. J..

[73] Gallardo J, Escalona-Noguero C, Sot B (2020). Role of α-synuclein regions in nucleation and elongation of amyloid fiber assembly. ACS Chem. Neurosci..

[74] Stephens AD (2020). Extent of N-terminus exposure of monomeric alpha-synuclein determines its aggregation propensity. Nat. Commun..

[75] Afitska K, Fucikova A, Shvadchak VV, Yushchenko DA (2017). Modification of C terminus provides new insights into the mechanism of α-synuclein aggregation. Biophys. J..

[76] Ranjan P, Kumar A (2017). Perturbation in long-range contacts modulates the kinetics of amyloid formation in α-synuclein familial mutants. ACS Chem. Neurosci..

[77] Bertoncini CW (2005). Familial mutants of alpha-synuclein with increased neurotoxicity have a destabilized conformation. J. Biol. Chem..

[78] Mizuguchi C (2019). Mechanisms of aggregation and fibril formation of the amyloidogenic N-terminal fragment of apolipoprotein A-I. J. Biol. Chem..

[79] Buell AK (2014). Solution conditions determine the relative importance of nucleation and growth processes in α-synuclein aggregation. Proc. Natl. Acad. Sci. U.S.A..

[80] Conway KA (2000). Acceleration of oligomerization, not fibrillization, is a shared property of both α-synuclein mutations linked to early-onset Parkinson's disease: Implications for pathogenesis and therapy. Proc. Natl. Acad. Sci. U.S.A..

[81] Winner B (2011). In vivo demonstration that alpha-synuclein oligomers are toxic. Proc. Natl. Acad. Sci. U.S.A..

[82] Kamiyoshihara T, Kojima M, Uéda K, Tashiro M, Shimotakahara S (2007). Observation of multiple intermediates in alpha-synuclein fibril formation by singular value decomposition analysis. Biochem. Biophys. Res. Commun..

[83] Lázaro DF (2014). Systematic comparison of the effects of alpha-synuclein mutations on its oligomerization and aggregation. PLoS Genet..

[84] Camino JD (2021). The role of water in the primary nucleation of protein amyloid aggregation. Biophys. Chem..

[85] Zimmermann MR (2021). Mechanism of secondary nucleation at the single fibril level from direct observations of Aβ42 aggregation. J. Am. Chem. Soc..

[86] Saric A (2016). Physical determinants of the self-replication of protein fibrils. Nat. Phys..

[87] Farzadfard A (2022). The C-terminal tail of α-synuclein protects against aggregate replication but is critical for oligomerization. Commun. Biol..

[88] Guerrero-Ferreira R, Kovacik L, Ni D, Stahlberg H (2020). New insights on the structure of alpha-synuclein fibrils using cryo-electron microscopy. Curr. Opin. Neurobiol..

[89] Kumari P (2021). Structural insights into alpha-synuclein monomer–fibril interactions. Proc. Natl. Acad. Sci. U.S.A..

[90] Prymaczok NC, Riek R, Gerez J (2016). More than a rumor spreads in Parkinson's disease. Front. Hum. Neurosci..

[91] Terada M (2018). The effect of truncation on prion-like properties of α-synuclein. J. Biol. Chem..

[92] Sorrentino ZA (2018). Physiological C-terminal truncation of α-synuclein potentiates the prion-like formation of pathological inclusions. J. Biol. Chem..

[93] Ohgita T (2021). Novel conformation-selective monoclonal antibodies against apoA-I amyloid fibrils. FEBS J..

[94] Cohlberg JA, Li J, Uversky VN, Fink AL (2002). Heparin and other glycosaminoglycans stimulate the formation of amyloid fibrils from alpha-synuclein in vitro. Biochemistry.

[95] Nath S, Meuvis J, Hendrix J, Carl SA, Engelborghs Y (2010). Early aggregation steps in alpha-synuclein as measured by FCS and FRET: Evidence for a contagious conformational change. Biophys. J..

[96] Nielsen L (2001). Effect of environmental factors on the kinetics of insulin fibril formation: Elucidation of the molecular mechanism. Biochemistry.

