# Supplementary material for: Mechanisms of enhanced aggregation and fibril formation of Parkinson’s disease-related variants of α-synuclein
Source: Sci Rep. 2022 Apr 26;12:6770. doi: 10.1038/s41598-022-10789-6 (PMC9043213; doi:10.1038/s41598-022-10789-6)
Supplement: Supplementary file 1 — Supplementary Information. [file 41598_2022_10789_MOESM1_ESM.pdf]

Supplementary Information

Mechanisms of enhanced aggregation and fibril formation of Parkinson's disease-related variants of α-synuclein

Takashi Ohgita[1, *], Norihiro Namba[1], Hiroki Kono[1], Toshinori Shimanouchi[2], Hiroyuki Saito[1]

[1] Department of Biophysical Chemistry, Kyoto Pharmaceutical University, 5 Nakauchi-cho, Misasagi, Yamashina-ku, Kyoto 607-8414, Japan

[2] Graduate School of Environmental and Life Science, Okayama University, Okayama 700-8530, Japan

This supplementary data consists of: Figures S1–S4

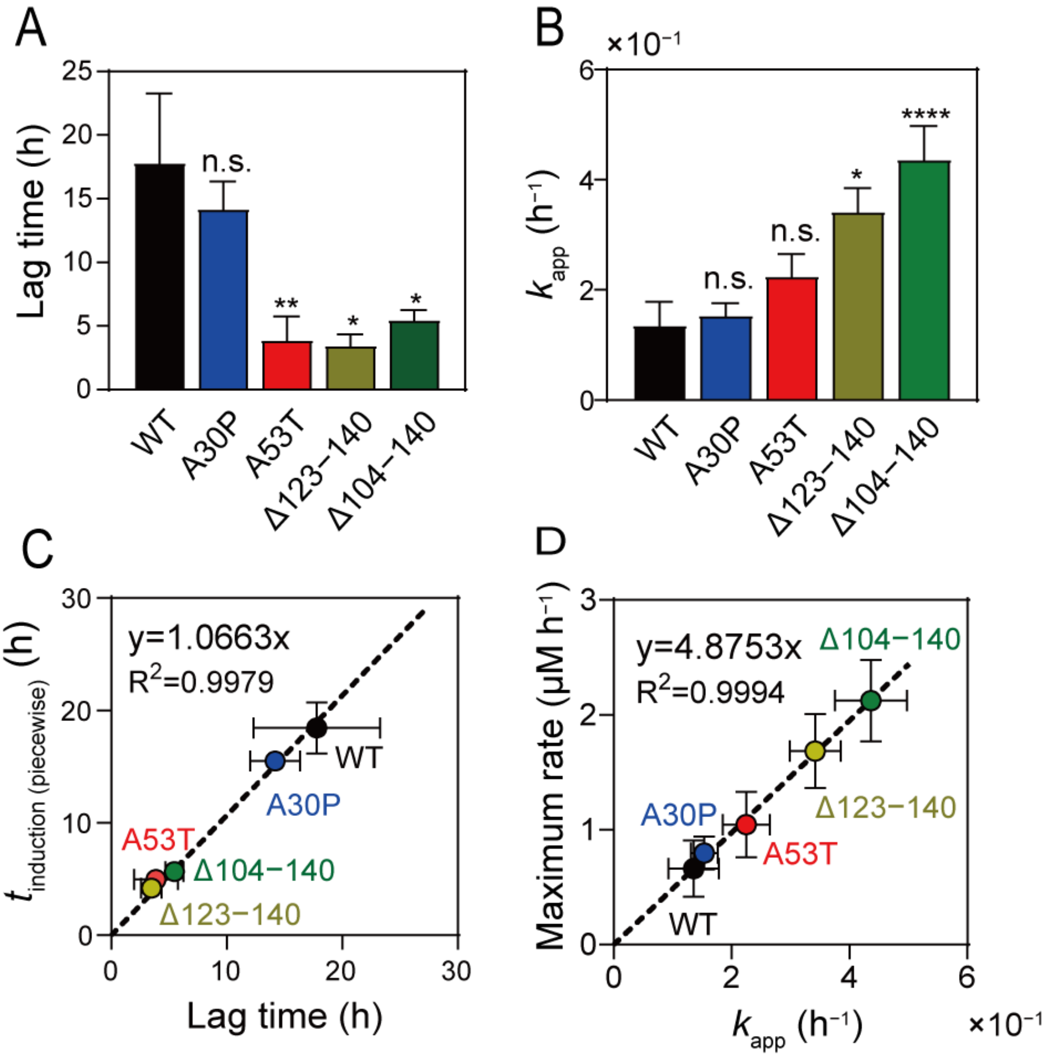

Figure S1. Kinetic parameters for fibril formation of α-syn variants obtained by sigmoidal fitting to ThT fluorescence traces. *A* and *B*, Comparison of lag time (A) and apparent rate constant for fibril growth k_{app} (B) in fibril formation of α-syn variants shown in Fig. 2A. The values of k_{app} were determined according to sigmoidal equation 1. Lag times were calculated from half time and k_{app} according to lag time = (half time) − 2/ k_{app}. *Error bars* represent S.E. *, p<0.05; **, p<0.01; ****, p<0.0001 versus WT. n.s., not significant. *C* and *D*, Correlations between lag time and $t_{induction\ (piecewise)}$ (C) or k_{app} and maximum rate (D) obtained according to the sigmoidal equation 1 and the Finke-Watzky 2-step model equation 2. *Error bars* represent S.E. The *dashed lines* are linear regression lines. The equations and r-square values are also shown.

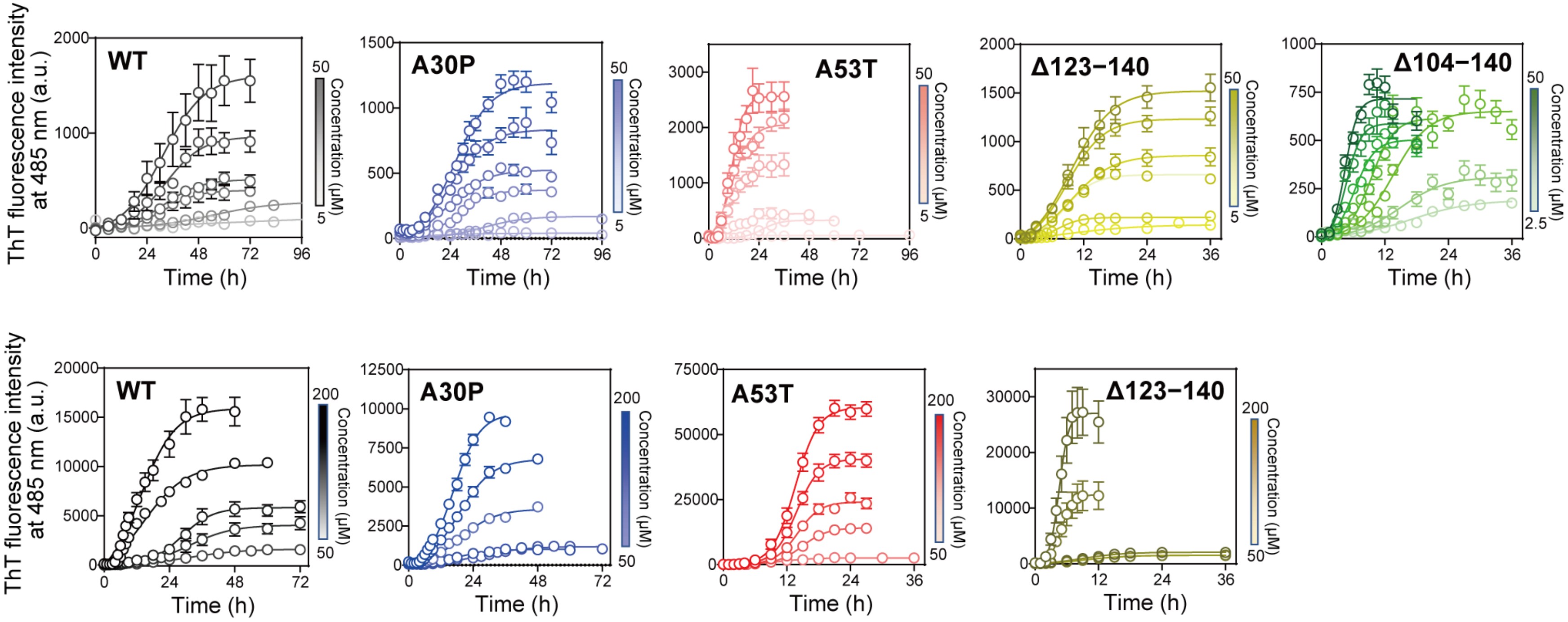

Figure S2. ThT fluorescence curves of α-syn variants with varying initial monomer concentration. The initial monomer concentration of α-syn was varied in the range of 5–200 μM for WT, A30P, A53T, and Δ123–140; 2.5–50 μM for Δ104–140. *Upper and lower panels* represent the curves in the concentration ranges of 2.5–50 and 50–200 μM of α-syn, respectively. *Error bars* represent S.E.

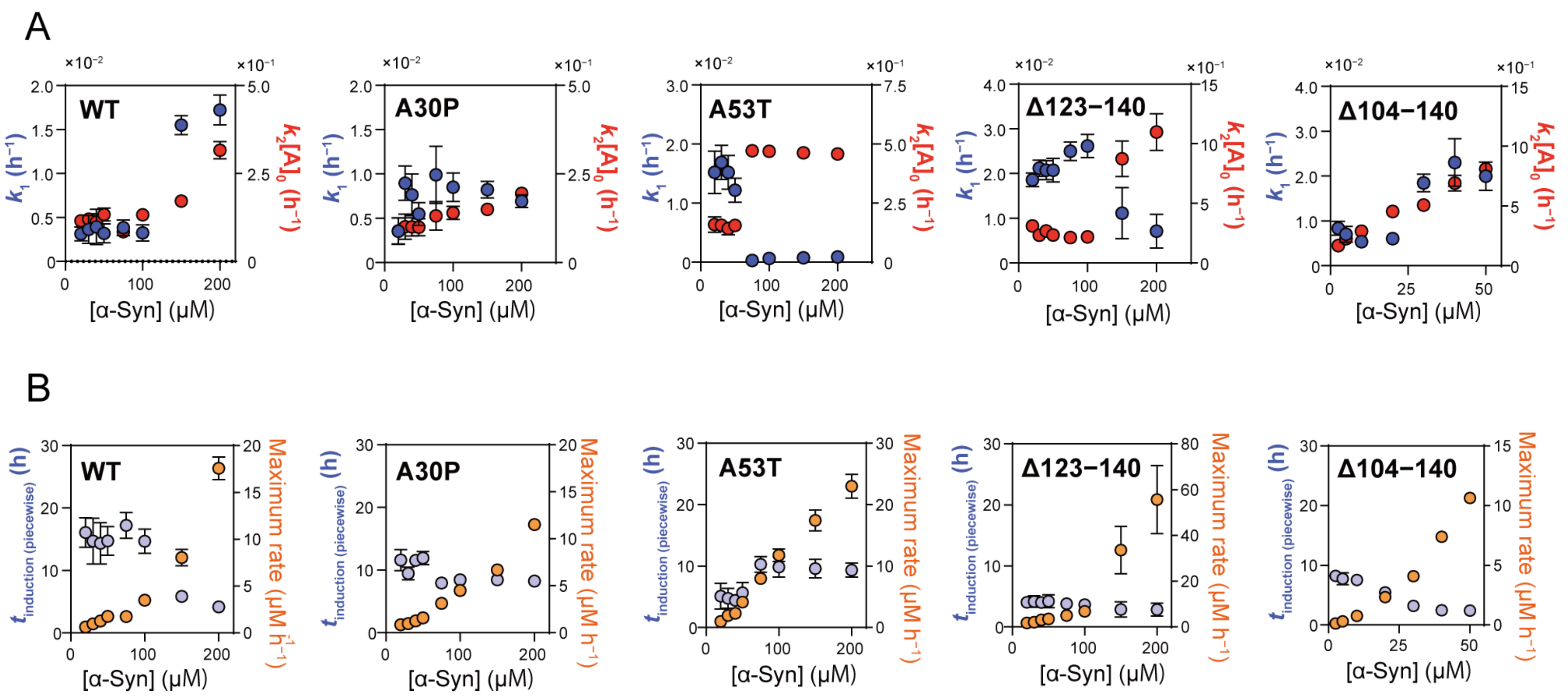

Figure S3. Finke-Watzky kinetic parameters for fibril formation of α-syn variants at different initial monomer concentrations. *A*, Rate constants for nucleation (k_1, blue) and fibril growth (k_2[A]$_0$, red). *B*, $t_{\text{induction (piecewise)}}$ (light blue) and maximum rate (orange) calculated from k_1 and k_2 values according to equations 3 and 4, respectively. *Error bars* represent S.E.

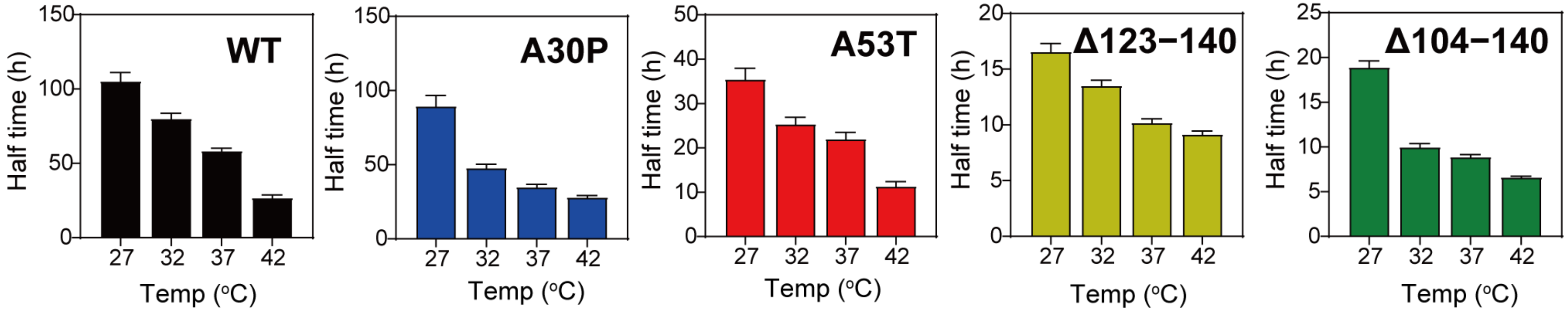

Figure S4. Half times for fibril formation of α-syn variants at different temperatures. The half time was determined according to sigmoidal equation 1. *Error bars* represent S.E.
